# Supplementary material for: Association between oral microbiome and seven types of cancers in East Asian population: a two-sample Mendelian randomization analysis
Source: Front Mol Biosci. 2023 Nov 21;10:1327893. doi: 10.3389/fmolb.2023.1327893 (PMC10702768; doi:10.3389/fmolb.2023.1327893)
Supplement: Supplementary file 1 [file DataSheet1.ZIP › Supplementary Materials/MR plots for tongue/tongue═╝/Ovarian cancer/pheno.1948_to_ovarian cancer_leave_one_out.pdf]

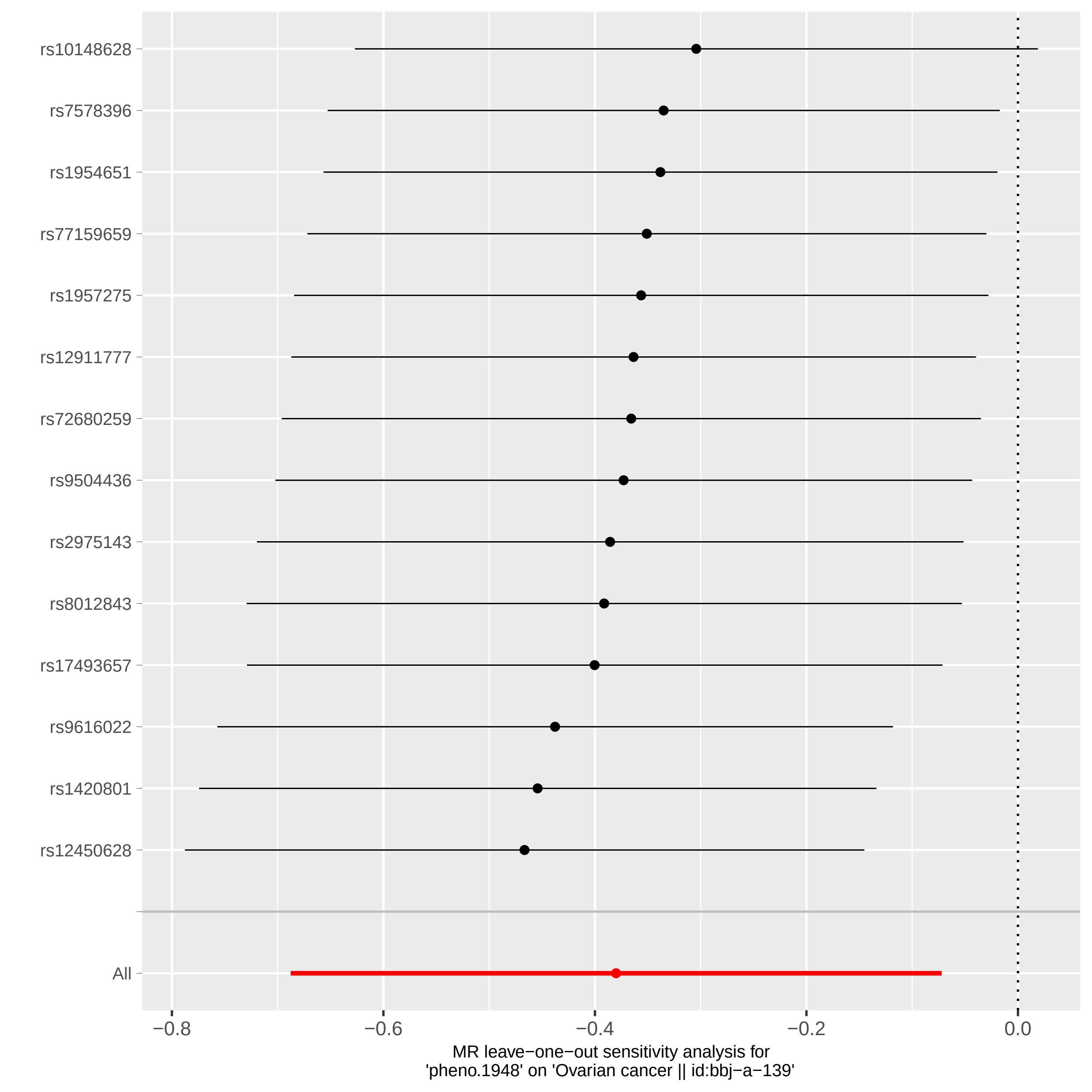

rs10148628
rs7578396
rs1954651
rs77159659
rs1957275
rs12911777
rs72680259
rs9504436
rs2975143
rs8012843
rs17493657
rs9616022
rs1420801
rs12450628
All
−0.8
−0.6
−0.4
−0.2
0.0
MR leave−one−out sensitivity analysis for
'pheno.1948' on 'Ovarian cancer || id:bbj−a−139'
